# Assessment of frailty in cirrhosis using bedside measures and its correlation with Child-Turcotte-Pugh, MELD & MELD-Na Scores

**DOI:** 10.12669/pjms.38.5.5545

**Published:** 2022

**Authors:** Bader Faiyaz Zuberi, Tazeen Rasheed, Faiza Sadaqat Ali, Nimrah Bader, Rabia Sadaf

**Affiliations:** 1 Bader Faiyaz Zuberi FCPS, Meritorious Professor, Department of Medicine/Gastroenterology, Dow Medical College, Dow University of Health Sciences, Karachi, Pakistan; 2 Tazeen Rasheed FCPS, Assistant Professor, Department of Medicine/Gastroenterology, Dow Medical College, Dow University of Health Sciences, Karachi, Pakistan; 3 Faiza Sadaqat Ali FCPS, Senior Registrar, Department of Medicine/Gastroenterology, Dow Medical College, Dow University of Health Sciences, Karachi, Pakistan; 4 Nimrah Bader MD, Geriatric Medicine Fellow, University of Miami/Jackson Health System, Miami, FL, USA; 5 Rabia Sadaf FCPS, Consultant, Dr Ruth KM Pfau Civil Hospital, Karachi, Pakistan

**Keywords:** Liver Frailty Index, Sarcopenia, Cirrhosis, Hand Grip Strength, Chair to Stand Time, Three Position Balance

## Abstract

**Objectives:**

To assess frailty in cirrhotic by calculating Liver Frailty Index (LFI) using bedside clinical tests and correlate it with Child-Turcotte-Pugh (CTP), MELD, MELD Na, Fib- 4 and Transient Elastography (TE) scores

**Methods:**

This cross-sectional observational comparative study was carried out in Dr Ruth KM Pfau Civil Hospital, Karachi from August 2020 to September 2021. Patients were subjected to three performance-based testing including dominant hand grip strength (HGS), Chair to Stand (CTS) Time & Three Position Balance (TPB). LFI was calculated using the online LFI calculator, available at http://liverfrailtyindex.ucsf.edu and classified as ‘Robust’ if LFI <3.2, ‘Prefrail’ LFI between 3.2 and 4.4, and ‘Frail’ as LFI ≥4.5. Correlation of frailty with MELD, MELD-Na and CTP Scores was done. Means of MELD & MELD-Na Scores and CTP scores were calculated in all 3 classes of frailty using one way ANOVA. A *p*-value of ≤.05 was taken as significant.

**Results:**

Out of 118 patients, 62 (52.5%) were males. Mean MELD score was 11.4 ±3.3, MELD-Na was 15.97 ±8.54, CTP 8.25 ±2.21, Fib-4 was 2.79 ±1.034 and TE score was 18.20 ±9.17. Mean LFI was 3.87 ±1.07; mean HGS was 18.12 ±4.68; mean of CTS was 9.62 ±3.55. LFI Class distribution in our cohort showed Robust were 36 (30.5%), Prefrail 34 (28.8%) and Frail were 48 (40.8%). Correlation of all these variables with LFI showed significant correlation with LFI, but highest correlation coefficient was seen with MELD-Na.

**Conclusion:**

Significant correlation between frailty score in cirrhotic with cirrhosis severity scores highlights the need for frequently assessing LFI in all cirrhotic at regular follow up visits.

## INTRODUCTION

Frailty/sarcopenia has gained importance due to their strong association with adverse clinical outcomes in patients of cirrhosis and transplantation. Frailty is the pivotal feature of physical decline in aging and incapacitating diseases, it is defined as vulnerability to health stressors leading to physical dependency and death.^1^ Sarcopenia, which routinely accompanies frailty, is the anatomic reduction of muscle mass, develops as a result of disproportion of muscle protein integration and disintegration.^2^ Although both are assessed differently, frailty and sarcopenia are closely coupled notions that share common clinical outcomes. Frailty is a critical determinant of health outcomes in almost every disease and assessing overall health of patients is an important tool for clinical decision makings. Lai and colleagues describe frailty as, a patient’s vulnerability to stress and decreased physiologic reserve, is considered as a major contributing factor in patient outcome.^3^ After adjusting for standard biochemical indices such as MELD-Na, frailty of patient play an important role in the prognosis. Originally frailty concept was coined in the geriatric population and was later adopted and validated in cohorts of patients with decompensated cirrhosis.^3^,^4^ Liver Frailty Index (LFI), consisting of three performance-based tests (grip, chair stands, balance).^5^

Comprehension that frailty is possibly modifiable, is the temptation to recognise and formulating some interventions, that can fetter its progress in cirrhotic. As per author’s knowledge, there is no local data highlighting frailty and its index in cirrhotic as well as no clinical practice guideline to offer evidence-based management for frailty, so this study aimed to document this modifiable complication in cirrhotic, using liver frailty index and to assess its association with currently available prognostic scores like MELD & CTP scores. This study can help in making some foundation for future research on improving frailty, hence survival benefit in cirrhotic patients. The objectives was to determine frailty in cirrhosis of liver using Liver Frailty Index (LFI) and to correlate it with CTP, MELD & MELD-Na Scores.

*Operational Definitions: Cirrhosis: was defined if two of the following criteria were met*

Transient Elastography (TE) value by

Fibroscan of >14.0 kPa

Fib-4 Score of >3.25^3^

***FIB-4 was calculated with following formula:***


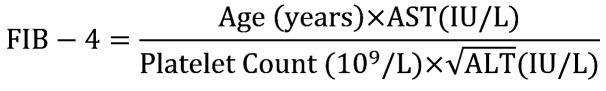


### MELD Calculation Formulas:

“MELD = 3.78 × Log_e_ [serum bilirubin (mg/dL)] + 11.2 × Log_e_ [INR] + 9.57 × Log_e_ [serum creatinine (mg/dL)] + 6.43.^6^

MELD-Na = MELD + 1.59 x (135-Na^+^), with maximum and minimum Na^+^ values of 135 and 120 mEq/L, respectively.^7^ Higher the MELD-Na score, higher was the severity of chronic liver disease.” Details of points allocation for Child-Turcotte-Pugh Score is given in Table-I.^8^

**Table I:** Child-Turcotte-Pugh (CTP) Score Calculation.^8^

Clinical & Biochemical Measurements	Points scored for increasing abnormality		

	1	2	3
HE Grade	Absent	1-2	3-4
Ascites	Absent	Slight	Moderate
Bilirubin	< 2.0	2.0-3.0	> 3.0
Albumin	> 3.5	2.8-3.5	< 2.8
PT Prolongation (INR)	< 4 (<1.7)	4-6 (1.7-2.3)	> 6 (>2.3)

### Liver Frailty Index (LFI):

Liver Frailty Index was calculated using the online LFI calculator available at http://liverfrailtyindex.ucsf.edu.^9^ It consists of four variables, one fixed variable gender, and three performance based variables dominant hand grip strength measured by hand dynamometer, time to do five chair stands & holding three position balance. Calculations were done as per following formula:

“LFI = (–0.330 × sex-adjusted grip strength) + (–2.529 × number of chair stand per second) + (–0.040 × balance time) + 6.”^10^

### Frailty Classification:

“The classifications of frailty were determined by using previously established cut-offs of the LFI with ‘Robust’ defined as LFI <3.2, ‘Prefrail’ defined as LFI between 3.2 and 4.4, and ‘Frail’ defined as LFI ≥4.5.”^5^

### Hand Grip Strength (HGS):

Hand grip strength testing was done using hand dynamometer in dominant hand. Patient was asked to squeeze the device with full force/strength that he/she can apply and reading on display was noted. The procedure was repeated after one minute for two more times and all three values was recorded for analysis. Value of <26 kg in males and <18 kg in females in dominant hand was taken as decreased.^11^

### Chair to Stand Time (CTS):

Test was administered using a chair without arms with rubber tips placed on its legs chair was not secured against wall. Seat height of chair was 17 inches. Patient initially sat in middle of chair with foot apart of the approximate width of shoulder and knees are slightly more flexed form neutral position so they at floor about 10 cm posteriorly. Arms held at chest and crossed at wrists.^12^ Patients were instructed to stand up and sit down five times continuously without pause as quickly as they can on GO. Time was measured in seconds using standard stopwatch from GO till the buttocks touch the chair after 5^th^ repetition.^13^

### Three Position Balance (TPB):

Three position balance includes maintaining balance in three positions, i.e., Side Balance, Semi Tandem Balance & Tandem Balance. Patient was asked to maintain balance for each position for 10 seconds. Patient can hold out arms or move body to maintain balance but without moving feet for each position. If feet are moved, test is terminated, and time noted for duration of position maintained in seconds using a stopwatch. Each position was demonstrated to the patient by investigator. Investigator will stand near the patient to help if patient tends to sway/fall.

**Fig.1:**
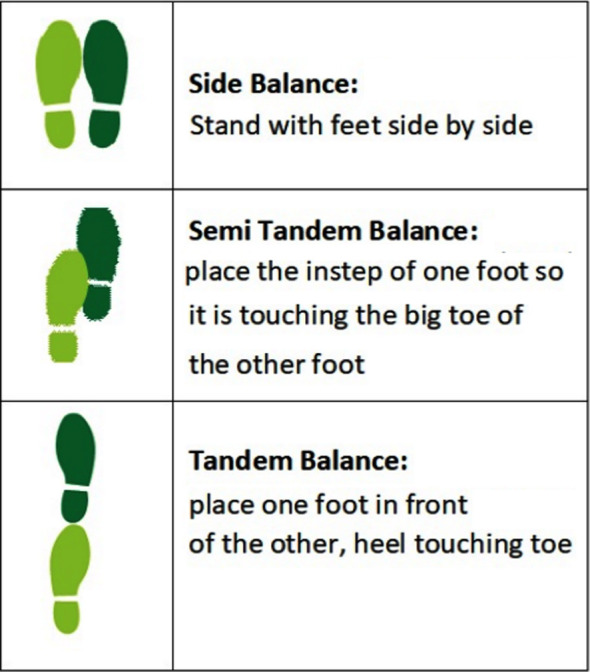
Three Position balance.

## METHODS

This study was conducted in Medical Unit I, Dr Ruth KM Pfau Civil Hospital, Karachi, Pakistan during the period of August 2020 and September 2021. Sample size calculation was done using PASS version 19.03 software using reported frailty in cirrhosis of 35.3%.^13^ A sample size of 118 achieves 90.16% power to detect a difference (P1-P0) of -0.147 using a two-sided exact test with a significance level (alpha) of 0.05. Study has approval from IRB of DUHS vide letter # IRB-1613/DUHS/Approval/2020/140 dated 8^th^ August 2020. Patients were included after informed consent.

### Inclusion Criteria:

Patients of cirrhosis defined in operational definition of either gender with age 18-70 years were included.

### Exclusion Criteria:

Patients with heart failure, diabetes mellitus, pleural effusion, any malignancy, stroke, diagnosed cases of myopathy, polymyositis, arthritis, and hepatic encephalopathy grades three and four were excluded.

### Data collection procedure:

Selected patients underwent detail clinical examination. Blood was withdrawn for testing for bilirubin, AST, albumin, creatinine, serum sodium and INR. All patients were subjected to three performance-based testing including dominant hand grip strength measured by hand dynamometer, time to do 5 chair stands & holding 3 position balance. Results was recorded in proforma. Liver Frailty Index was calculated using the online LFI calculator and patients was classified as Robust, prefrail or frail based on LFI.

### Data analysis procedure:

Demographic data of selected patients was reported including means ±SD was reported for all scale variables. Distribution of scale variables was checked for normality by Kolmogorov-Smirnov Test. Frequencies were reported for gender, HE Grades, Ascites Grades and Frailty Class and their frequencies were compared by χ^2^ test. MELD and MELD-Na calculations were done and recorded. Correlation of frailty with MELD, MELD-Na and CTP Scores was done using Kendall’s tau-b Test. Means of these variables were also compared on basis of LFI Class using ANOVA. A *p*-value of ≤.05 was taken as significant. Statistical analysis was done by IBM SPSS Statistics for Windows, Version 26.0. (Armonk, NY: IBM Corp.).

## RESULTS

Out of 118 patients, 62 (52.5%) were males and 56 (47.5%) were females. Mean age of females was 42.36 ±6.4 and mean age of males was 41.98 ±5.98, making no difference in age among gender *t* (116) = 0.327, *p* = .744. Mean MELD score was 11.4 ±3.3, Meld-Na was 15.97 ±8.54, CTP 8.25 ±2.21, Fib-4 was 2.79 ±1.034 and TE score was 18.20 ±9.17. Mean albumin was 3.22 ±.33 mg/dl and mean platelets was 113.59 ±27.81. Details of baseline characteristics with comparison on basis of gender is given in Table-II. No significant differences were found in these parameters on basis of gender on Student’s t-test. Hepatic encephalopathy Grade 1 was present in 35 (29.7%) while Grade 2 was present in 30 (25.4%) patients. Patients of Grade 3 & 4 hepatic encephalopathy were excluded as per selection criteria. Mild ascites was present in 26 (22%) while moderate ascites was present 37 (31.4%) patients.

**Table II:** Comparison of Means of baseline characteristics of patients according to gender with Student’s t-test.

	Gender						

	Mean	SD	Female		Male		p-value

			Mean	SD	Mean	SD	
MELD Score	11.14	3.31	11.06	3.04	11.22	3.57	.787
MELD-Na Score	15.97	8.54	15.12	8.11	16.73	8.91	.308
CTP Score	8.25	2.21	8.11	2.22	8.39	2.21	.494
Fib-4 Score	2.79	1.03	2.82	.93	2.76	1.13	.791
TE (kPa)	18.20	9.17	19.57	7.67	16.97	10.25	.119
Albumin (mg/dl)	3.22	.33	3.16	.38	3.28	.28	.067
Platelets (x10^9/L)	113.59	27.81	116.27	26.19	111.18	29.21	.320
Bilirubin (mg/dl)	1.64	1.07	1.55	.93	1.72	1.18	.397
ALT (U/L)	55.47	33.08	53.25	14.45	49.47	18.24	.607
AST (U/L)	51.26	16.59	53.75	26.90	56.90	37.97	.218
INR	1.64	.53	1.59	.46	1.68	.59	.396
Na (mmol/l)	132.01	5.41	132.32	4.94	131.73	5.83	.549
Creatinine (mg/dl)	1.71	.72	1.72	.67	1.70	.77	.903

Significant Level ≤.05.

Mean LFI was 3.87 ±1.07; mean HGS was 18.12 ±4.68; mean of 5-time CTS was 9.62 ±3.55. Comparisons were done for HGS, CTS and TPB on basis of gender and presented in Table-III. Except for HGS no significant difference was present on Student’s t-test. LFI Class distribution in our cohort showed Robust were 36 (30.5%), Prefrail 34 (28.8%) and Frail were 48 (40.8%).

**Table III:** Mean Frailty test scoring and comparison based on gender.

	Gender						

	Mean	SD	Female		Male		p-value

			Mean	SD	Mean	SD	
HGS	18.12	4.68	17.23	4.64	18.93	4.60	.048
CTS (sec)	9.62	3.55	9.0	3.3	10.2	3.7	.057
TPB Side	8.45	1.36	8.61	1.51	8.31	1.21	.238
TPB Semi Tandem	8.36	1.38	8.57	1.50	8.18	1.25	.122
TPB Tandem	8.18	1.45	8.39	1.58	7.98	1.31	.131
LFI	3.87	1.08	3.72	1.05	4.01	1.09	.145

*Significant Level ≤.05.

Means of MELD, MELD-Na, CTP, Fib-4 & TE Scores were compared by LFI Class using one-way ANOVA, all variables showed significant differences in values on basis of LFI Class, details are given in Table-IV. Correlation was also tested of all these variables with LFI using Kendall’s tau-b test and all variables showed significant correlation with LFI, but highest correlation coefficient was seen with MELD-Na. Details are given in Table-V.

**Table IV:** Mean of patients variables with LFI class using One Way ANOVA.

	LFI Class	N	Mean	SD	p-value
MELD Score	Robust	36	8.97	1.25	<.001
	Prefrail	34	9.55	1.87	
	Frail	48	13.90	3.24	
MELD-Na Score	Robust	36	8.96	1.19	<.001
	Prefrail	34	9.87	2.11	
	Frail	48	25.54	4.39	
CTP Score	Robust	36	6.78	1.35	<.001
	Prefrail	34	8.23	1.86	
	Frail	48	9.37	2.32	
Fib-4 Score	Robust	36	2.33	0.47	<.001
	Prefrail	34	2.50	1.26	
	Frail	48	3.33	0.92	
TE (kPa)	Robust	36	13.64	7.15	<.001
	Prefrail	34	17.03	10.53	
	Frail	48	22.46	7.61	

Significant Level ≤.05.

**Table V:** Correlation of LFI with MELD, MELD-Na, Fib-4 & TE Scores with LFI by Kendall’s tau-b Test.

		MELD Score	MELD-Na Score	CTP Score	Fib-4 Score	TE Score (kPa)
LFI (n=118)	Correlation Coefficient	.558**	.779**	.247**	.247**	.268**
	*p*-value	<.001	<.001	<.001	<.001	<.001

** . Correlation is significant at the 0.01 level (2-tailed).

## DISCUSSION

Art of medicine lies in assessing global assessment of patient’s health by clinician. Relying too heavily on eye ball testing for global health assessment for clinical decision is always debated because of lack of reproducibility as naked eye may miss certain subtle signs for prognosis like slight slowing of gate speed^14^ or progressive decline in muscle mass.^15^ Objective bed side tool for assessing global health of patient by clinician with good reproducibility in clinical decision making and prognosis is much needed. In this study we evaluated interesting, feasible, bedside objective clinical tool of physical decline that is Liver Frailty Index, in cirrhotic patients and compared this with other available tools of prognosis in cirrhosis. This is the first study that assessed Liver frailty index in cirrhotic patients, highlighting strong positive correlation of LFI with CTP, Meld-Na, Meld, Fib-4 and fibro scan scores, making it a reliable tool of physical decline for global health assessment of patients with cirrhosis.

A hospitalized patient of cirrhosis is at increased risk of mortality due to his underlying disease.^16^ The tools available for risk assessment (e.g., MELD) incompletely capture the magnitude of disease, while investigational tools (e.g., APACHE) require expertise and are not accepted by most clinicians.^17^ MELD score has limitation that it does not include parameters to gauge patient’s frailty. Interesting to note that with MELD score of 15, one patient may carry out all routine activity with well controlled ascites, while another patient may not even stand without support from his refractory ascites, sarcopenia and physical frailty. Although while documenting their prognosis and decision for listing on transplantation, these two patients have the equal (relatively low) predicted probability of mortality based on their MELD score – and therefore, the same (low) preference for liver transplantation – but any clinician by his eye ball testing can easily predict that the latter patient clearly bears a higher risk of death. Yet at the present time, we lack the authentic tools to objectively capture this risk. MELD score is now replaced with MELD-Na which also takes into account of hyponatremia in these patients and has been shown much superior in predicting mortality as compared to MELD.^18^,^19^

Frailty measures functionality of patients and it follows sarcopenia, which is the measure of anatomical reduction in muscle mass. As sarcopenia advances, so does the frailty. Sarcopenia has been authenticated to predict mortality in cirrhotic, waiting for transplant, & encephalopathy in cirrhotic patients.^15^,^20^ Frailty can be considered to have same rather more prognostic implications as does sarcopenia and it has more merits over sarcopenia and other prognostic scores. Firstly, measuring frailty index is much easier than sarcopenia which requires advanced imaging for diagnosis.^15^ Second, cognitive deterioration that could limit performance, reflect untreated or undertreated Hepatic Encephalopathy (HE), is not a part of calculating LFI, as compare to Child-Turcotte-Pugh (CTP) Score. Third, and most conclusively, frailty is a changeable risk factor that can be improved with rigorous nutritional aid and physiotherapy.

This interesting component of improvement in frailty has been shown in many studies, demonstrating that exercise improves physical frailty in terms of aerobic capacity, sarcopenia and quality of life in chronic liver disease and after liver transplantation.^21^ Supervised aerobic exercises like treadmill or cycle ergometer, done at least twice weekly for up to eight weeks, significantly improved aerobic capacity in cirrhotic patients, in terms of VO_2_ peak, i.e., the volume of oxygen that the body can utilize during physical exertion (+1.7 to 5.3 mL/kg/min; *p* < .05), muscle mass and reduced fatigue.^22^

One major strength of our study is that we included all patients of cirrhosis following inclusion criteria, not just those waiting for liver transplantation. Whereas majority of studies evaluated LFI in cirrhotic awaiting transplantation and did not enrol patients <60 years if their MELD scores were <12.^9^ So their studies cannot be generalized to all cirrhotic patients. We did a prospective study on large number of patients, whereas some previous studies evaluated frailty on retrospective data.^23^ Furthermore the tools which we used to calculate frailty were easy to perform, less time consuming and bed side tests that include both upper limb ( HGS) and lower limbs ( CTS time) as well as balance (TPB), hence can help in global health assessment of cirrhotic patients.

Limitations: One of the limitations of our study is that we excluded patients with grade 3 and 4 hepatic encephalopathy due to their inability to perform frailty test. This may introduce spectrum bias in our results. On the other hand, we included 35 patients (29.7%) grade1, 30 patients (25.4%) with Grade-2 encephalopathy, 26 patients (22.0%) with mild ascites and 37 patients (31.4%) with moderate ascites. Although patients with hepatic encephalopathy grade 1-2 and patients with ascites may have had difficulty with performing the frailty tests, we felt that their lower scores would truly reflected their decrease muscle mass and poor nutritional status, rather than reflect an inability to follow the frailty test instructions.

## CONCLUSION

Significant correlation between frailty score in cirrhotic with cirrhosis severity scores highlights the need for frequently assessing LFI in all cirrhotic at regular follow up visits. Frailty being a modifiable factor of physical decline, if improved can result in significant improvement in Meld, Meld Na and CTP score, hence overall survival of patients.

### Authors’ Contribution:

**BFZ, TR, NB:** Substantial contributions to conception and design, or acquisition of data, or analysis and interpretation of data.

**FSA, RS:** Drafting the article or revising it critically for important intellectual content.

**BFZ:** Final approval of the version to be published.

**TR, NB:** Statistical Analysis.

Agreement to be accountable for all aspects of the work in ensuring that questions related to the accuracy or integrity of any part of the work are appropriately investigated and resolved (All Authors).
